# Hypotensive and Vasorelaxant Effects of Sericin-Derived Oligopeptides in Rats

**DOI:** 10.1155/2013/717529

**Published:** 2013-11-07

**Authors:** Amnart Onsa-ard, Dawan Shimbhu, Jiraporn Tocharus, Manote Sutheerawattananonda, Rungusa Pantan, Chainarong Tocharus

**Affiliations:** ^1^Department of Biochemistry, Faculty of Medical Science, Naresuan University, Phitsanulok 65000, Thailand; ^2^Department of Physiology, Faculty of Medicine, Chiang Mai University, Chiang Mai 50200, Thailand; ^3^School of Food Technology, Institute of Agricultural Technology, Suranaree University of Technology, Nakhon Ratchasima 30000, Thailand; ^4^Department of Anatomy, Faculty of Medicine, Chiang Mai University, Chiang Mai 50200, Thailand

## Abstract

Sericin-derived oligopeptides obtained from silk cocoons were investigated for the *in vivo* hypotensive effect and investigated for the underlying mechanism involved in vasodilation in isolated rat thoracic aorta. In normotensive anesthetized rats, oligopeptides induced an immediate and transient hypotensive activity. In rat aortic rings, oligopeptides induced a concentration-dependent vasorelaxation in vessels precontracted with both KCl and phenylephrine (PE) with endothelium-intact or endothelium-denuded rings. In endothelium-intact rings, pretreatment with N**ω**-Nitro-L-arginine methyl ester hydrochloride (L-NAME, 100 *µ*M), an inhibitor of the NO synthase (NOS) or 1H-[1,2,4]oxadiazolo[4,3-a]quinoxalin-1-one (ODQ, 1 *µ*M), a selective inhibitor of the guanylyl cyclase enzyme, significantly reduced the relaxant effect of oligopeptides. However, indomethacin, an inhibitor of the cyclooxygenase, had no effect on oligopeptides-induced relaxation. In addition, pretreatment with tetraethylammonium (TEA, 5 mM) reduced the maximal relaxant effect induced by oligopeptides. By contrast, relaxation was not affected by 4-aminopyridine (4-AP, 1 mM), glibenclamide (10 *µ*M), or barium chloride (BaCl_2_, 1 mM). In depolarization Ca^2+^-free solution, oligopeptides inhibited calcium chloride- (CaCl_2_-) induced contraction in endothelium-denuded rings in a concentration-dependent manner. Nevertheless, oligopeptides attenuated transient contractions in Ca^2+^-free medium containing EGTA (1 mM) induced by 1 *µ*M PE, but they were not affected by 20 mM caffeine. It is obvious that potent vasodilation effect of oligopeptides is mediated through both the endothelium and the vascular smooth muscle.

## 1. Introduction

Peptides derived from dietary proteins have been determined to modulate physiological functions. Bioactive peptides have also elicited antihypertensive and vasodilator functions [1]. The most common underlying mechanism of bioactive peptides has been studied in angiotensin-converting enzyme (ACE) inhibition [2–4]. Although the ACE inhibitor effects of bioactive peptides are most often studied, the vasorelaxant activity has also shown an important evidence for lowering blood pressure. It is well established that dietary protein found in soy protein [5], milk casein [6], and sweet potato [7] and its hydrolysates are capable of reducing blood pressure and modulating vascular activity. Recently, there are many reports that dietary-protein-derived peptides lower blood pressure and modulate vasodilation from several sources including egg white [8], soy protein [9], and *κ*-casein [10]. Moreover, silk fibroin, a core protein of silk fiber, has been shown to have potential hypotensive and antioxidant actions by fibroin hydrolysate [11]. Silk-fibroin-derived dipeptides, such as glycine-tyrosine, also produce an antihypertensive effect through angiotensin-converting enzyme (ACE) inhibition [12].

Sericin is a glue protein consisting of 20%–30% of the total cocoon weight which is synthesized in the middle gland of the *Bombyx mori* silkworm. Sericin is insoluble in cold water and is an indigestible intestinal protein. Sericin is composed of 18 amino acids and contains protein in a wide range of molecular weights from 10 to over 300 kDa [13]. Sericin has shown numerous bioactivities, such as antioxidant [14], antitumor [15], antiproliferation [16], and anticholesterolemic properties [17]. Sericin can be degraded into peptides or hydrolysate forms. However, sericin and its hydrolysates have not been reported for vasorelaxation and blood pressure lowering. Therefore, in the present study, we investigated the possible mechanism involved in the blood pressure-lowering and vasomodulating effects of sericin-derived oligopeptides.

## 2. Materials and Methods

### 2.1. Preparation of Sericin-Derived Oligopeptides

Silk cocoons were autoclaved for 30 min to dissolve sericin protein. The sericin-rich protein solution was filtered through a cheese cloth to separate the extracted cocoons from the liquid part. The sericin solution obtained was subjected to enzymatic hydrolysis by protease (from *Bacillus* species, 16 unit/g, EC no. 2327522, Sigma, St. Louis, MO, USA). One mL of protease enzyme solution (0.01 unit/mL protease enzyme in 0.036 M CaCl_2_ solution at a 1 : 1 volumetric ration) was added to 300 mL of the obtained sericin solution and incubated under shaking conditions at 37°C for 1 h. The solution was then heated to 90°C for 15 min to stop the enzymatic activity and cooled to room temperature before centrifugation at 9500 ×g for 15 min at 4°C to separate the solid portions. Oligopeptides with a molecular weight lower than 5 kDa were separated from larger oligopeptides by ultra membrane filtration using a hollow fiber membrane with 5000 MWCO (molecular weight cutoff) (GE Healthcare Bio-Sciences AB, Uppsala, Sweden). The oligopeptides solution obtained was freeze-dried and kept in a sealed container at room temperature until use.

### 2.2. Experimental Animals

Male Wistar rats (200–250 g) were obtained from the National Laboratory Animal Center, Mahidol University, Salaya, Nakornpathom, Thailand. All animals were housed under a 12:12 h light-dark cycle conditions, with maintained temperature (24 ± 1°C). The animals were allowed free access to rodent diet and tap water. The experiment protocol was approved by the Animal Ethics Committee in accordance with the guide for the care and use of laboratory animals prepared by Chiang Mai University.

### 2.3. Blood Pressure in Anesthetized Normotensive Rats

The rats were anesthetized by intraperitoneal injection of sodium pentobarbital (50 mg/kg BW). The femoral artery was cannulated with polyethylene tubing 50 (Clay-Adams PE-50) filled with 100 IU of heparin/mL connected to a pressure transducer to measure blood pressure. The blood pressure signal was amplified and converted to a digital signal by a bridge amplifier coupled with PowerLab (ADInstruments, Sydney, Australia). Systolic blood pressure (SBP), diastolic blood pressure (DBP), and heart rate (HR) were recorded with LabChart 7 software (ADInstruments, Sydney, Australia). Oligopeptides were administered via a cannula inserted into the femoral vein with similar tubing to facilitate the intravenous injection of oligopeptides (0.1 *μ*g–1000 *μ*g/kg BW). Animals were allowed to equilibrate at least 20 min before the administration of any drugs [18]. Arterial pressure was allowed to return to the baseline level before the subsequent injections were conducted. 

### 2.4. Record of Isometric Vascular Tone

#### 2.4.1. Preparation of Isolated Rat Thoracic Aortic Ring

The rats were anesthetized by intraperitoneal injection of sodium pentobarbital (50 mg/kg BW). The thoracic aorta was immediately removed and cleaned from the connective tissues and fat. Aortic rings (3–5 mm) were obtained and immediately immersed into ice-cold Kreb's solution (composition in mM: NaCl 122; KCl 5; (HEPES) 10; KH_2_PO_4_ 0.5; NaH_2_PO_4_ 0.5; MgCl_2_ 1; glucose 11; CaCl_2_ 1.8; pH 7.4) until use. The endothelium-denuded rings were obtained by mechanically removing the endothelial layer by gentle rubbing around the internal vascular surface [19]. The aortic rings were mounted between a pair of platinum wires in an organ bath containing Krebs' solution, maintained at 37°C and continuously bubbled with air. A resting tension of 1 gram was applied to each tissue and allowed to equilibrate at least 1 h. The endothelium integrity was verified by relaxation to Ach (1 *μ*M) in rings precontracted by PE (1 *μ*M). The vascular endothelium was considered intact when aortic rings were relaxed 90% of the PE-induced precontractions, whereas the endothelium-denuded ring was confirmed with an absence of vasorelaxation. Changes in isometric tension were recorded and analyzed through a force transducer (Iworx Systems, Inc., NH, USA), coupled with bridge amplifier (ADInstruments, Sydney, Australia), PowerLab (ADInstruments, Sydney, Australia), and signal virtualization by Labchart 7 software (ADInstruments, Sydney, Australia).

#### 2.4.2. Effect of Oligopeptides on Contractions Induced by PE and K^+^

Vasorelaxant effects of oligopeptides were investigated in both endothelium-intact and endothelium-denuded aortic rings. After the ring was preequilibrated, the aortic ring was precontracted with PE (1 *μ*M) or KCl (80 mM) until the stability of tension was developed and followed by cumulative exposure to oligopeptides at the concentration of 0.001–10 mg/mL. The extent of relaxation was expressed as the percentage of PE- or KCl-induced contraction.

#### 2.4.3. Role of Endothelium-Derived Mediators in the Relaxant Effect of Oligopeptides

To investigate the mechanism responsible for oligopeptides-induced vasorelaxation, the endothelium-intact rings were precontracted with PE (1 *μ*M) for 30 min after being exposed to either L-NAME (100 *μ*M), an inhibitor of the NO synthase (NOS), indomethacin (10 *μ*M), an inhibitor of the cyclooxygenase (COX), or L-NAME plus indomethacin. And, then, vasorelaxation was carried out by the cumulative exposure to oligopeptides at the concentrations of 0.001–10 mg/mL.

#### 2.4.4. Role of K^+^ Channels Involvement in Oligopeptides-Induced Vasorelaxation

To examine the role of K^+^ channels involvement in vasorelaxation, the endothelium-denuded ring was used for this determination by preincubation with one of the following K^+^ channel blockers: tetraethylammonium, a nonselective inhibitor of K^+^ channels (TEA, 5 mM), 4-aminopyridine, an inhibitor of voltage-operated K^+^ channel (4-AP, 1 mM), glibenclamide, an inhibitor of K^+^ channels activated by adenosine triphosphate (10 *μ*M), and BaCl_2_ (1 mM) for 30 min before PE (1 *μ*M) precontraction. Then, the cumulative concentration response of oligopeptides at the concentrations of 0.001–10 mg/mL was directly added.

#### 2.4.5. Role of Soluble Guanylyl Cyclase (sGC) in Oligopeptides-Induced Vasorelaxation

To evaluate whether oligopeptides possessed vasorelaxation via the sGC activation, the endothelium-intact ring was incubated with 1H-[1,2,3]oxadiazolo[4,3-*α*]quinoxalin-1-one, a selective inhibitor of the guanylyl cyclase enzyme (ODQ, 1 *μ*M) for 30 min before contraction with PE (1 *μ*M) treatment. Oligopeptides at the concentrations of 0.001–10 mg/mL were cumulatively added. The ability of vasorelaxation was compared in absence (control) and presence of ODQ.

#### 2.4.6. Effect of Oligopeptides on Extracellular Ca^2+^ Influx to SMC

To investigate the effects of oligopeptides on Ca^2+^ channel, the endothelium-denuded aortic ring was placed under Ca^2+^-free Krebs' solution for 20 min and then exposed for an additional 10 min to K^+^ (80 mM), Ca^2+^-free solution for complete smooth muscle cell depolarization to open a voltage-operated Ca^2+^ channel (VOCC). The cumulative concentration-response curve of CaCl_2_ (ranging from 10 *μ*M to 10 mM) was obtained. After the maximal response was performed, the rings were washed out and replaced with Ca^2+^-free solution for 20 min. The Ca^2+^-free 80 mM K^+^ was reexposed to flowing with preincubation of either oligopeptides (1, 3, 5, and 10 mg/mL) or nifedipine, L-type Ca^2+^ channel blocker (1 *μ*M) for 20 min. The maximal contraction obtained with the control concentration response curve to CaCl_2_ was taken as 100%, and all values were calculated as a percentage of the maximal response.

#### 2.4.7. Effect of Oligopeptides on Ca^2+^ Release from Intracellular Stores Sensitive to PE and Caffeine

To investigate the effect of oligopeptides on PE- or caffeine-sensitive intracellular calcium stores, endothelium-denuded rings were bathed in Ca^2+^-free Kreb's solution containing 1 mM EGTA after incubation with 80 mM KCl for successful Ca^2+^ loading into SR. The transient contractions were obtained in aortic rings by PE (1 *μ*M) or caffeine (20 mM) in Ca^2+^-free solution before and after being incubated with oligopeptides (1, 5, and 10 mg/mL). The results were expressed as percentage of the response induced by PE or caffeine alone.

### 2.5. Statistical Analysis

All data were expressed as mean ± SEM. Statistical analysis was performed using Student's *t*-test or one-way analysis of variance (ANOVA) followed by Tukey's post hoc test. The *P* < 0.05 was considered to be significant. Concentration-response curves were plotted, and experimental data were obtained by using nonlinear curves fit program (GraphPad Prism 5).

## 3. Results

### 3.1. Hypotensive Effect of Oligopeptides in Normotensive Anesthetized Rats

**Figure 1:**
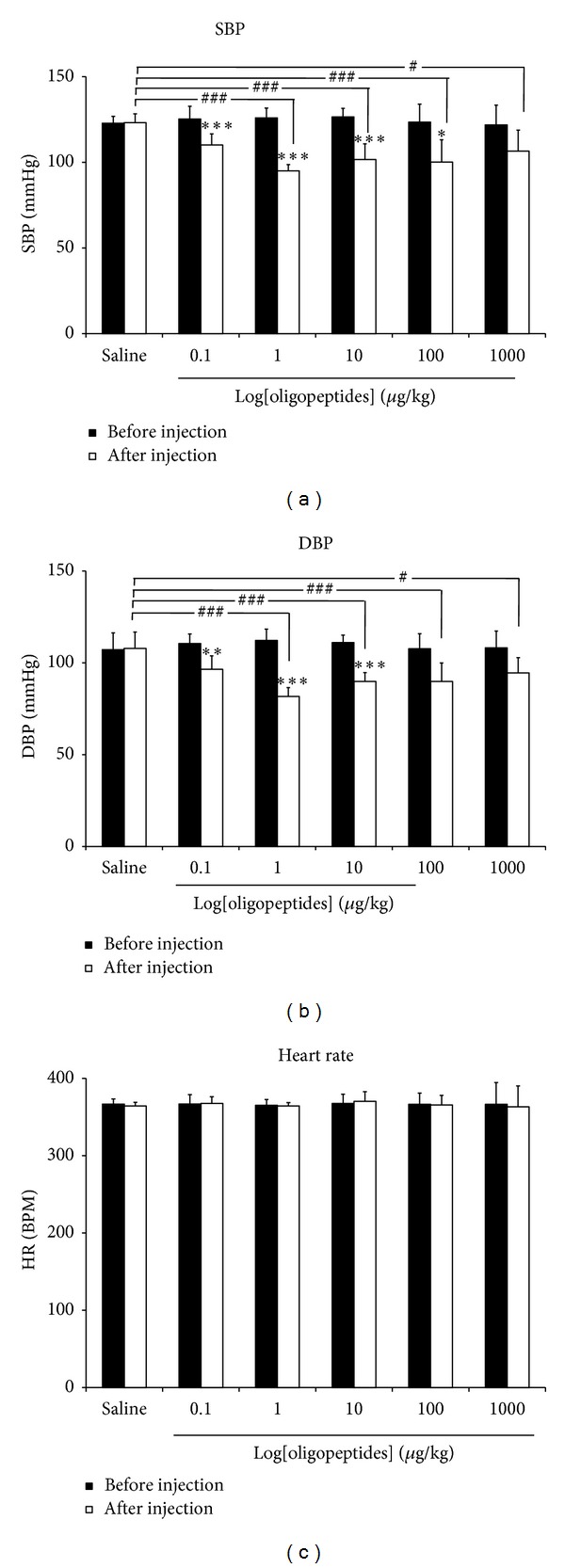
The maximal reduction of (a) systolic blood pressure, (b) diastolic blood pressure, and (c) heart rate caused by intravenous administration of oligopeptides (1–1000 *μ*g/kg BW) in normotensive rats. The results are expressed as mean ± SEM; *n* = 6; *P < 0.05; **P < 0.01; and ***P < 0.001 compared with before injection. ^#^ *P* < 0.05, and ^###^ *P* < 0.001 compared with saline injection.

We investigated the effect of oligopeptides on blood pressure in normotensive rats. The baselines of SBP, DBP, and HR were 122.8 ± 1.64 mmHg, 107 ± 3.74 mmHg, and 366 ± 2.67 BPM, respectively. Intravenous administration of oligopeptides dose dependently decreased SBP and DBP in rats (Figures 1(a) and 1(b)). The hypotensive response in each dose of oligopeptides was completely recovered to the baseline within a few minutes. In addition, oligopeptides at all doses did not show any obvious effect on HR (Figure 1(c)).

### 3.2. Effect of Oligopeptides on Contraction Induced by PE and KCl

**Figure 2:**
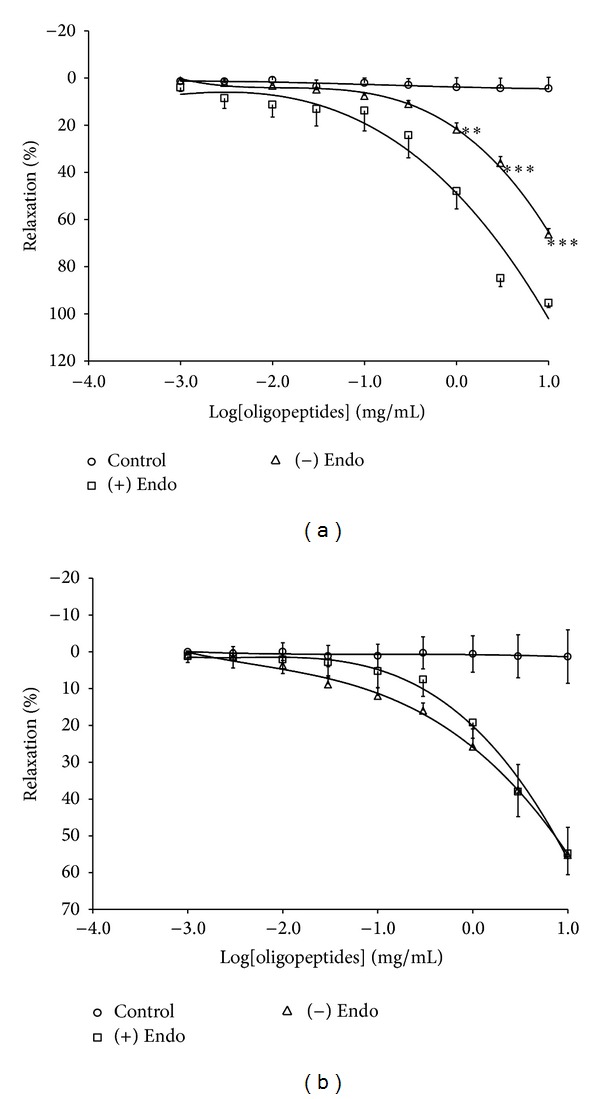
Vasorelaxant effects of oligopeptides on the contraction induced by PE (a) and 80 mM KCl (b) in endothelium-intact and -denuded aortic rings. The results are expressed as mean ± SEM; *n* = 6; **P < 0.01; and ***P < 0.001 compared with endothelium-intact rings. +Endo = endothelium-intact rings, and −Endo = endothelium-denuded rings.

Oligopeptides significantly relaxed PE- and 80 mM KCl-precontracted aortic rings in a concentration-dependent manner. However, oligopeptides completely relaxed in PE- (*E*_max⁡_ = 95.46 ± 1.86%) induced contractions in the aortic rings and partially relaxed in KCl- (80 mM, *E*_max⁡_ = 55.22 ± 7.56%) induced contractions (Figures 2(a) and 2(b)). The endothelium-denuded ring significantly decreased the vasodilator effects only on the PE precontraction (Figure 2(a)), while, for 80 mM KCl, precontraction had no effects (Figure 2(b)).

### 3.3. Role of Nitric Oxide (NO) and Prostanoids in Oligopeptides-Induced Vasorelaxation

**Figure 3:**
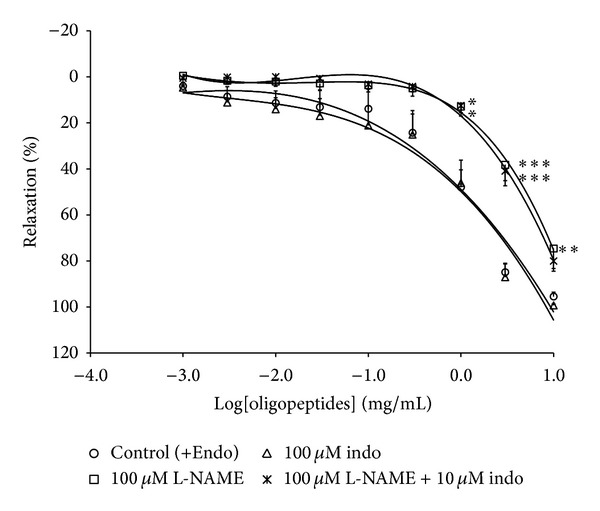
Concentration-response curve of oligopeptides on vasorelaxation modulated by L-NAME (100 *μ*M, *n* = 6), Indo (10 *μ*M, *n* = 8), and combination of L-NAME and Indo (*n* = 6) in PE- (1 *μ*M) precontracted aortic rings with endothelium. The results are presented as mean ± SEM; *P < 0.05; **P < 0.01; and ***P < 0.001 compared with control.

NO and prostacyclin are known to be vasorelaxing mediators derived from endothelium. In endothelium-intact preparation, oligopeptides relaxed the PE- (1 *μ*M) induced contractions with an EC_50_ value of 1.86 ± 0.16 mg/mL (Figure 3). In the presence of L-NAME and L-NAME plus indomethacin, the relaxant effect of oligopeptides against PE- (1 *μ*M) induced contraction was markedly attenuated with an EC_50_ value of 13.49 ± 0.18 and 8.71 ± 0.15 mg/mL, respectively (Figure 3). By contrast, treatment of the aortic ring with indomethacin showed no modification in endothelium-intact rings (Figure 3).

### 3.4. Role of K^+^ Channel in Oligopeptides-Induced Vasorelaxation

**Figure 4:**
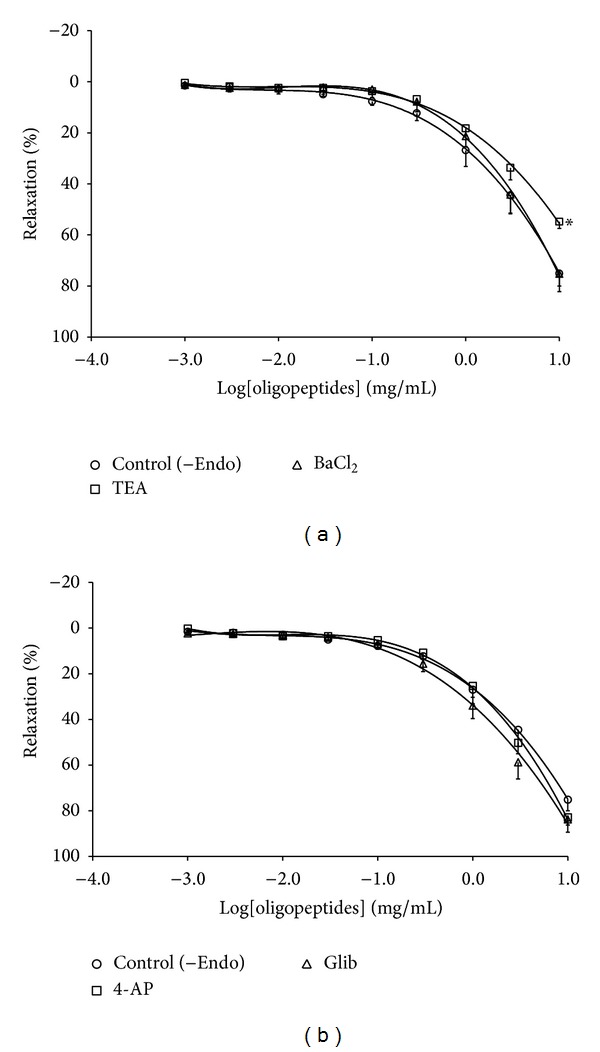
Effects of inhibitor of K^+^ channels on vasorelaxant response induced by oligopeptides on endothelium-denuded aortic rings pre-contracted with PE (1 *μ*M) in presence of (a) TEA (5 mM, *n* = 7) and BaCl_2_ (1 mM, *n* = 6) and (b) 4-AP (1 mM, *n* = 7) and Glib (10 *μ*M, *n* = 6). The data are expressed as mean ± SEM, *P < 0.05, compared with control (−Endo).

To verify the role of K^+^ channel in oligopeptides-induced vasorelaxation, the endothelium-denuded ring was preincubated with different potassium channel inhibitors: tetraethylammonium (5 mM), 4-aminopyridine (1 mM), glibenclamide (10 *μ*M), or BaCl_2_ (1 mM) before the ring was contracted with PE. All inhibitors did not alter the concentration-response curve for oligopeptides as shown in Figures 4(a) and 4(b). However, the maximal relaxant activity of oligopeptides which were preincubated with tetraethylammonium was markedly reduced (*E*_max⁡_ = 54.82 ± 2.63%) compared to the control (*E*_max⁡_ = 83.74 ± 5.61%).

### 3.5. Role of sGC in Oligopeptides-Induced Vasorelaxation

**Figure 5:**
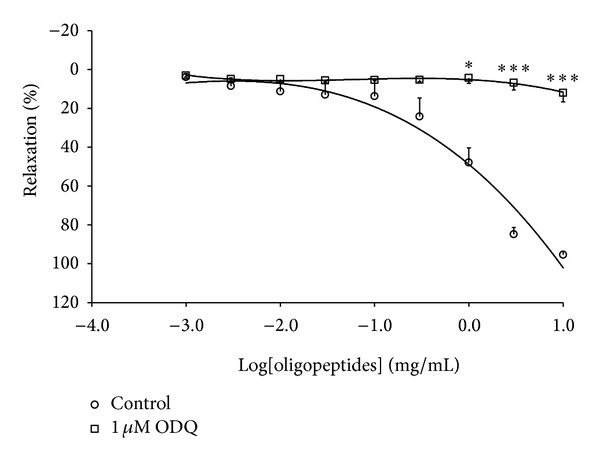
Vasorelaxant response of oligopeptides on rat aortic rings precontracted by PE (1 *μ*M) in presence of ODQ (1 *μ*M, *n* = 6). Results are presented as mean ± SEM; *n* = 6; *P < 0.05; ***P < 0.001 compared with control.

ODQ (1 *μ*M) significantly abolished the oligopeptides-induced vasorelaxation in the endothelium-intact ring (Figure 5). It markedly decreased both the potency (EC_50_ = 11.22 ± 0.91 mg/mL) and maximal relaxation (*E*_max⁡_ = 12.09 ± 4.63%) when compared to untreated control values (EC_50_  1.86 ± 0.79 mg/mL, and *E*_max⁡_ = 95.03 ± 1.86%).

### 3.6. Effects of Oligopeptides on CaCl_2_-Induced Contractions

**Figure 6:**
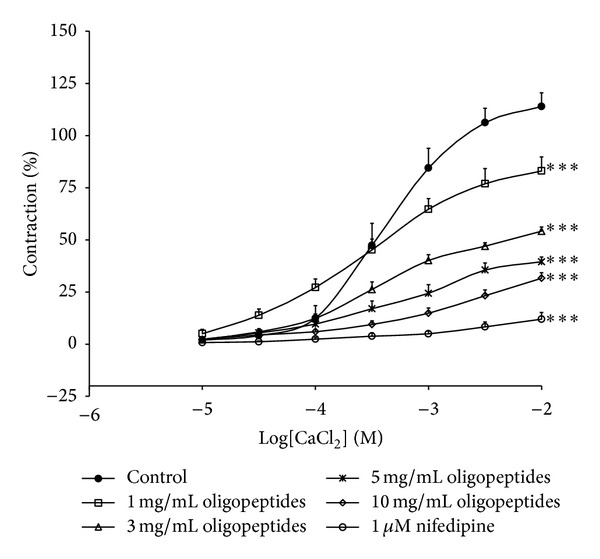
Inhibitory effects of oligopeptides (1, 3, 5, and 10 mg/mL) or nifedipine (1 *μ*M) on the concentration-response curve for CaCl_2_-induced contraction in Ca^2+^-free 80 mM KCl solution. Results are expressed as mean ± SEM; *n* = 6; *P < 0.05; **P < 0.01; and ***P < 0.001 compared with control.

The concentration-response curves of CaCl_2_ (10 *μ*M–10 mM) were performed in the 80 mM KCl Ca^2+^-free solution. The percentage of maximal response (*E*_max⁡_) of CaCl_2_ alone (100%) was attenuated in the presence of oligopeptides (1, 3, 5, and 10 mg/mL) in a concentration-dependent manner (83.02 ± 6.78%, 54.00 ± 1.98%, 39.52 ± 2.28%, and 31.61 ± 2.6%, resp.), while EC_50_ values were similar (Figure 6). In addition, nifedipine (1 *μ*M) also abolished the contraction of CaCl_2_. This suggested that Ca^2+^ influx was probably reduced by oligopeptides.

### 3.7. Effect of Oligopeptides on the SR Ca^2+^ Release to PE and Caffeine Activation

**Figure 7:**
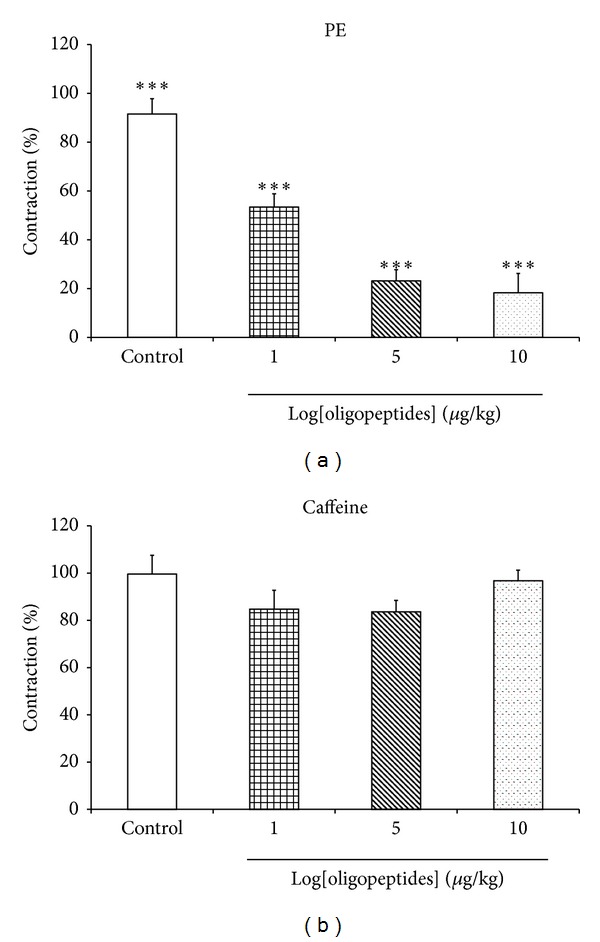
The effects of oligopeptides (1, 5, and 10 mg/mL) inhibited Ca^2+^ release from the SR by (a) PE (1 *μ*M) and (b) caffeine (20 mM) in isolated aortic rings without endothelium. Results are expressed as mean ± SEM; *n* = 6; ***P < 0.001 compared with control.

Preincubation of endothelium-denuded rings with oligopeptides at the concentrations of 1, 5, and 10 mg/mL dose dependently decreased the transient contractions induced by PE (10 *μ*M) in Ca^2+^-free Krebs' solution containing EGTA (1 mM) (Figure 7(a)). By contrast, oligopeptides produced no significant effect on the transient contractions induced by caffeine (20 mM) in a similar condition (Figure 7(b)).

## 4. Discussion

Sericin is a protein derived from the silk cocoon that appears as waste in silk processing. Sericin is a mixture of polypeptides of molecular mass varying between 10 and 300 kDa [13] and has high serine contents (30%–33%). In the past decade, various biological activities regarding sericin have been reported in gastric injuries [14] and protection against tumorigenesis [15], antioxidation, inhibition of tyrosinase [20], alcohol-induced liver [21], and UV-induced keratinocyte apoptosis [22]. In the present study, sericin was hydrolyzed, and it generated the bioactive peptides with low-molecular- (5 ≤ kDa) sized peptides. The sericin-derived oligopeptides exerted vasorelaxant activities in rats, which explain their hypotensive effects. We found that oligopeptides lowered blood pressure in normotensive rats. The reduction of blood pressure was transient, and it recovered to baseline level in a few minutes by dose-dependent intravenous administration of oligopeptides. Although the reduction of blood pressure occurred, the level of blood pressure was still within normotensive limits, whereas heart rate was unaffected. We further investigated the possible vasodilator effects of oligopeptides in the isolated rat aorta. Oligopeptides induced a concentration-dependent vasorelaxation on PE-induced precontractions in endothelium-intact and -denuded aortic rings, and this relaxation was attenuated by endothelium denudation. These results suggested that oligopeptides acted to cause vasorelaxation in endothelium-dependent aortic rings. The vascular endothelial cells pivotally acted in vascular homeostasis by modulating the vascular smooth muscle tone [23]. Thus, indomethacin, a cyclooxygenase inhibitor, and L-NAME, an inhibitor of NO synthase, were employed to investigate the involvement of prostacyclin and NO in vasodilation effect [24]. The results showed that oligopeptides-induced vasorelaxation was markedly suppressed by L-NAME, but not by indomethacin, suggesting the participation of NO in the vasorelaxation effect of oligopeptides. Furthermore, L-NAME plus indomethacin revealed no further inhibition than that observed with L-NAME alone. This showed that NO is a powerful relaxant mediator involvement in oligopeptides-induced vasorelaxation. 

It is well known that the NO/sGC pathway is one of the most common potential mechanisms that induce vascular smooth muscle relaxation through activation of guanylyl cyclase, leading to the accumulation of cyclic GMP that inhibits Ca^2+^ influx. ODQ, a soluble guanyly cyclase inhibitor, was used to confirm the relationship of the vasorelaxant response of oligopeptides. This finding showed the significant inhibitory effects of ODQ in oligopeptides-induced endothelium-dependent vasorelaxation, which confirmed that the vasorelaxation of the aorta elicited by oligopeptides was mediated in the endothelium-dependent NO/sGC/cGMP pathway. However, the vasorelaxation effect of oligopeptides was still observed in aortic endothelium-denuded rings, suggesting that oligopeptides have a direct effect on vascular smooth muscle cells. The opening of K^+^ channels or Ca^2+^ channel blocker in the vascular smooth muscle cells provides an important mechanism to dilate arteries. We further investigated the mechanisms of vasorelaxation that are independent of the endothelium. The vasorelaxant effect of oligopeptides was partially inhibited by the TEA (Ca^2+^-activated K^+^ channel blocker). Other K^+^ blockers, glibenclamide (ATP-sensitive K^+^ channel blocker), 4-AP (voltage-activated K^+^ channel blocker), and BaCl_2_ (inwardly rectifying K^+^ channel blocker) had no effects, suggesting that the relaxant response of oligopeptides was involved in the role of K^+^ channel opening of Ca^2+^-activated K^+^ channel in vascular smooth muscle cells. Influx of extracellular Ca^2+^ through voltage and/or receptor-operated calcium channels (VOCCs and/or ROCCs) plays an important role in vascular smooth muscle contraction. We note that oligopeptides induced vasorelaxation in aortic rings precontracted with KCl or phenylephrine. From this result, it can be concluded that oligopeptides induced vasorelaxation via different pathways. It is well known that KCl induces smooth muscle contraction through the activation of voltage-dependent calcium channels and subsequent release of calcium from sarcoplasmic reticulum, whereas phenylephrine-induced vasoconstriction is mediated by the stimulation of G-proteins coupled to alpha-adrenoceptors. We then investigated the mechanism for the vasodilator action of oligopeptides that could directly inhibit the Ca^2+^ influx in the vascular smooth muscle cells. Oligopeptides significantly reduced the contractile responseinduced by CaCl_2_ in the endothelium-denuded ring_,_ in concentration-dependent manner under a depolarizing solution. The contraction was abolished by nifedipine, a typical L-type voltage-operated calcium channel blocker, confirming the involvement of L-type voltage-operated calcium channels in the contractile response. The concentration-response curve of CaCl_2_ decreased in *E*_max⁡_, while the EC_50_ values were unchanged, suggesting that oligopeptides could act as a calcium channel blocker that interferes with Ca^2+^ influx through L-type Ca^2+^ channel of aorta smooth muscle membrane [25]. The influences of oligopeptides in Ca^2+^ released from intracellular stores were sensitive to phenylephrine, and caffeine was also determined. The oligopeptides markedly decreased the contractions induced by phenylephrine, which stimulates IP_3_-dependent Ca^2+^ release from the intracellular store [26]. By contrast, caffeine-induced contractions, which release Ca^2+^ from intracellular stores by IP_3_-independence, were not altered. Thus, it seems likely that the vascular effects of oligopeptides involved a reduction of IP_3_-dependent Ca^2+^ releases form SR sensitive to phenylephrine [27, 28]. 

Our results demonstrated that bioactive oligopeptides (≤5 kDa) fractions obtained from silk sericin lower blood pressure by a direct effect on both endothelium and vascular smooth muscle leading to vasodilation. Results from this study have also supported us to further identify and characterize bioactive peptides. 

## 5. Conclusions

This study demonstrated the hypotensive effect and vasorelaxant effect of silk sericin-derived oligopeptides on isolated rat aorta and the possible mechanisms. The results suggested that oligopeptides have a dose-dependent relaxing effect on the isolated rat aorta. The relaxing effect of oligopeptides is mediated through Ca^2+^ antagonism and the NO/sGC/cGMP pathway, which possibly explains the fall in BP. These findings provide scientific evidence supporting the therapeutic uses of sericin-derived oligopeptides as vascular modulators.
